# The effect of NGATHA altered activity on auxin signaling pathways within the Arabidopsis gynoecium

**DOI:** 10.3389/fpls.2014.00210

**Published:** 2014-05-21

**Authors:** Irene Martínez-Fernández, Sofía Sanchís, Naciele Marini, Vicente Balanzá, Patricia Ballester, Marisa Navarrete-Gómez, Antonio C. Oliveira, Lucia Colombo, Cristina Ferrándiz

**Affiliations:** ^1^Consejo Superior de Investigaciones Científicas - Instituto de Biología Molecular y Celular de Plantas (CSIC-UPV)Valencia, Spain; ^2^Department of Plant Sciences, Faculdade de Agronomia Eliseu Maciel, Plant Genomics and Breeding Center, Universidade Federal de PelotasPelotas, Brasil; ^3^Dipartimento di Biologia, Universita degli Studi di MilanoMilano, Italia

**Keywords:** gynoecium development, NGATHA, auxin synthesis, auxin transport, AUXIN RESPONSE FACTORS

## Abstract

The four *NGATHA* genes (*NGA)* form a small subfamily within the large family of B3-domain transcription factors of *Arabidopsis thaliana*. *NGA* genes act redundantly to direct the development of the apical tissues of the gynoecium, the style, and the stigma. Previous studies indicate that *NGA* genes could exert this function at least partially by directing the synthesis of auxin at the distal end of the developing gynoecium through the upregulation of two different *YUCCA* genes, which encode flavin monooxygenases involved in auxin biosynthesis. We have compared three developing pistil transcriptome data sets from wildtype, *nga* quadruple mutants, and a *35S::NGA3 line*. The differentially expressed genes showed a significant enrichment for auxin-related genes, supporting the idea of *NGA* genes as major regulators of auxin accumulation and distribution within the developing gynoecium. We have introduced reporter lines for several of these differentially expressed genes involved in synthesis, transport and response to auxin in *NGA* gain- and loss-of-function backgrounds. We present here a detailed map of the response of these reporters to *NGA* misregulation that could help to clarify the role of *NGA* in auxin-mediated gynoecium morphogenesis. Our data point to a very reduced auxin synthesis in the developing apical gynoecium of *nga* mutants, likely responsible for the lack of *DR5rev::GFP* reporter activity observed in these mutants. In addition, NGA altered activity affects the expression of protein kinases that regulate the cellular localization of auxin efflux regulators, and thus likely impact auxin transport. Finally, protein accumulation in pistils of several ARFs was differentially affected by *nga* mutations or *NGA* overexpression, suggesting that these accumulation patterns depend not only on auxin distribution but could be also regulated by transcriptional networks involving NGA factors.

## Introduction

The carpel is the female reproductive organ of the angiosperm flower and its most distinctive feature. Carpels typically occur at the center of the flower forming the gynoecium, most commonly fused into a single pistil (a syncarpic gynoecium) or less frequently as individual organs that collectively form an apocarpic gynoecium composed of several pistils. The gynoecium confers major advantages to flowering plants: provides protection for the ovules; enables pollen capture and pollen tube guidance and supports self- and inter-specific incompatibility; finally, after fertilization of the ovules, the gynoecium develops into a fruit, which protects the developing seeds and facilitates seed dispersal (Ferrandiz et al., 2010). To accomplish these roles, gynoecium development involves the differentiation of specialized functional modules: stigma forms at the apex of pistils to capture and germinate pollen grains; immediately below, the style is rich in transmitting tissues that conduct pollen tubes to the ovary, which is a basal structure that contains the ovules. In addition to these specialized tissues, other structures also develop in some pistils, such as those that will form the dehiscence zones in shattering fruits, or the septa that divide the ovary in locules (Sundberg and Ferrándiz, 2009; Ferrandiz et al., 2010).

To achieve differentiation and coordinated growth of the functional modules found in pistils, a suite of regulatory networks has to be in place. Most of our current knowledge on the major players in these networks comes from work carried out in *Arabidopsis thaliana*. A number of transcription factors have been identified with a role in the differentiation of the specialized tissues found in gynoecia or in the specification of polarity axes, and, while the picture is far from complete, we are now beginning to understand how their regulatory hierarchies and functional interactions work (reviewed in Balanzá et al., 2006; Ferrandiz et al., 2010). In addition to transcriptional regulation, the phytohormone auxin has been regarded as one of the major morphogens instructing gynoecium patterning and post-fertilization developmental events (Alabadi et al., 2009; Larsson et al., 2013). Local auxin maxima and minima have been shown to be instrumental for valve margin development and dehiscence (Sorefan et al., 2009). Most importantly, it is also known that auxin controls polarity in the apical-basal axis of the developing gynoecium. More than one decade ago, (Nemhauser et al., 2000) proposed a model for auxin-dependent distribution of tissues based on the phenotypes of *ettin (ett)* mutants, affected in the *AUXIN RESPONSE FACTOR 3* gene; the phenotypes of mutants defective in auxin transport such as *pinoid (pid)* or *pin-formed1 (pin1)*; and on the effects of inhibiting polar auxin transport (PAT) in gynoecium morphology. According to this model, auxin would be produced in the apical end of the pistil and transported basipetally, creating a gradient along the apical-basal axis that would be translated into the differentiation of the different functional modules: high apical auxin levels would direct the differentiation of style and stigma, intermediate levels would specify the ovary, and low basal levels, the gynophore. The Nemhauser model has been very useful to frame the role of different players in Arabidopsis carpel development, but conclusive proof of the proposed auxin gradient has never been obtained. Actually, detailed descriptions of auxin accumulation throughout gynoecium development using a *DR5rev::GFP* reporter have shown that auxin maxima are formed in the apical domain, first as isolated foci and later as a continuous apical ring, while the proposed gradient cannot be observed (Girin et al., 2011; Marsch-Martinez et al., 2012a; Larsson et al., 2013). In addition, several recent studies indicate that the dynamics of auxin accumulation, homeostasis and response within the developing gynoecium are highly complex and we are still far from fully comprehending how positional information is translated into developmental outputs in gynoecium differentiation (Sohlberg et al., 2006; Ståldal et al., 2008; Ståldal and Sundberg, 2009; Marsch-Martinez et al., 2012b). In any case, although detailed understanding of these mechanisms is still lacking, the pivotal role of auxin in apical-basal gynoecium patterning is widely acknowledged.

**Figure 1:**
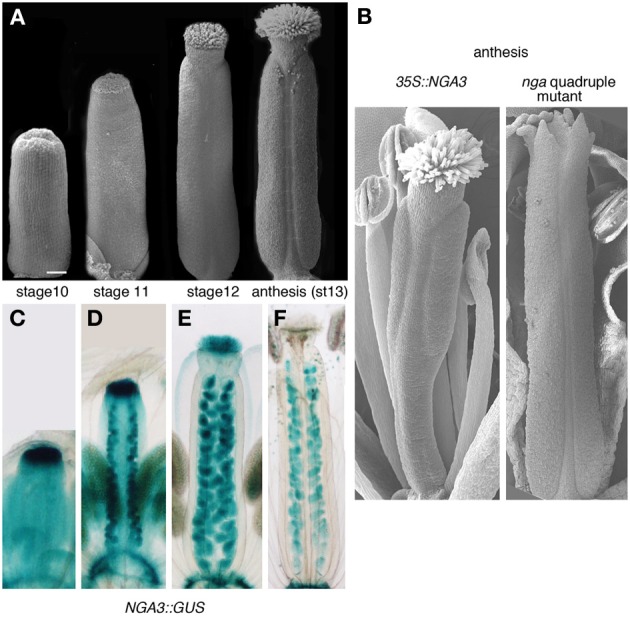
**Gynoecium development in Arabidopsis and NGA-related mutants. (A)** Scanning electron micrographs of wildtype gynoecia at different stages of development. **(B)** Phenotypes at anthesis of *35S::NGA3* (left) and *nga* quadruple mutant pistils (right). **(C–F)** Expression of *NGA3* in gynoecia at the same stages shown in **(A)**, revealed by the histochemical activity of a GUS reporter gene directed by *NGA3* regulatory sequences (Trigueros et al., 2009).

Among the transcriptional regulators directing carpel patterning, two small families of unrelated factors have been shown to be essential for apical tissue differentiation. The four *NGATHA* genes belong to the RAV clade of the large B3-domain transcription factor family and are redundantly required for the specification of style and stigma. *nga* quadruple mutants form apparently undisturbed ovaries but completely lack style and stigma, and the gynoecium ends apically as an open structure with several protrusions of valve-like tissue (Alvarez et al., 2009; Trigueros et al., 2009). Almost identical phenotypes are found in multiple mutants of the SHI/STY family of RING finger-like zinc finger motif transcription factors (Kuusk et al., 2006). *NGA* and *SHI/STY* genes also share similar expression patterns, which include the apical domain of developing gynoecia from stage 6 to stage 11–12, when style and stigma specification and differentiation take place (Figures 1C–F) (Kuusk et al., 2002; Alvarez et al., 2009; Trigueros et al., 2009). Interestingly, both NGA and SHI/STY factors have important connections to auxin. STY1 has been shown to directly regulate *YUCCA4 (YUC4)*, a gene encoding a flavin monooxygenase-like enzyme involved in auxin synthesis (Eklund et al., 2010). Likewise, *YUC4* and *YUC2* are not expressed in the gynoecium apex of lines where *NGA* genes were downregulated, a regulatory interaction that appears to be conserved also in other dicot species (Trigueros et al., 2009; Fourquin and Ferrandiz, 2014). Moreover, *NGA3* overexpression carpel phenotypes resemble the effects of PAT inhibition and of weak *ett* mutant alleles, and also affect other auxin-related processes in the plant, such as apical dominance, leaf morphology, or secondary root development, suggesting that *NGA* genes may interact with auxin signaling at multiple levels (Alvarez et al., 2009; Trigueros et al., 2009). In this study, we aim to characterize in detail the response of several components of the auxin signaling network to altered levels of NGA activity in the gynoecium, hoping to clarify the mechanisms of NGA action in auxin-mediated carpel morphogenesis.

## Materials and methods

### Plant material

*nga1-4* (line WiscDsLox429G06), *nga2-2* (line SM.20993) *nga3-3* (AMAZE En-1 line 6AAi79), and *nga4-3* (AMAZE En-1 line 6AAB133i) alleles were used to generate *nga* quadruple mutants. Genotyping was performed as previously described (Trigueros et al., 2009). All reporter lines used in this study have been previously described: *YUC8::GUS* (Rawat et al., 2009), *TAA1::GFP:TAA1* (Stepanova et al., 2008), *AMI1::GUS* (Hoffmann et al., 2010), *DR5rev::GFP* (Benková et al., 2003), *PID::GUS*, *PID::PID:GFP* (Lee and Cho, 2006), *WAG2::GUS* (Santner and Watson, 2006), *PIN3::PIN3:GFP* (Lee and Cho, 2006), *ARF8::GUS*, *ARF11::ARF11:GFP, ARF18::ARF18:GFP* (Rademacher et al., 2011).

### RNAseq analysis

Arabidopsis carpels between stages 8–13 from wildtype, *nga* mutant and *35S::NGA3* plants were collected manually from 15 developing inflorescences and stored transiently in cold extraction buffer of the Qiagen RNA extraction kit. RNA extraction was made with the Qiagen RNA extraction kit. RNA was analyzed for yield and quality on a Bioanalyzer 2100 (Agilent 2100). Libraries for sequencing were prepared from 2–4 μg total RNA using Illumina TruSeq RNA kits and sequenced with Illumina HiSeq2000. Quality control on the raw sequence data was done using FastQC (Babraham Bioinformatics). Reads were aligned to whole genome sequences from the TAIR10 A. thaliana database (www.arabidopsis.org) and analyzed using the CLC Genomics workbench (www.clcbio.com). RPKM (reads per kilobase per million) was considered as expression values. Two biological replicates for wildtype and three for *nga* and *35S::NGA3* were used for sequencing. After normalization, Bagerley's test and a FDR correction were used for statistical analysis of samples. Genes with a corrected FDR *p*-value < 0.05 and with a fold change >1.4 or <-1.4 were selected for gene ontology analysis with the agriGO toolkit (Du et al., 2010).

### Reporter activity detection

GUS histochemical detection was performed as previously described (Trigueros et al., 2009).

For GFP detection, fluorescent images were captured using an LSM 780 confocal scanning laser inverted microscope (Zeiss). GFP was excited using a 488 nm line of an argon ion laser. GFP emission spectra were collected between 500–550 nm and plastid autofluorescence was collected between 601 and 790 nm.

### Auxin micro-application

For the micro-application experiment, 80 mg of indolacetic acid (IAA) (Sigma, St. Louis, MO, USA) were dissolved in 2 mL of ethanol. IAA, or ethanol for mock treatment, was added to 10 gr of lanolin containing 2.5% liquid paraffin. The lanolin paste was applied to the apical end of stage 8–10 gynoecia using plastic pipette tips under a dissecting microscope, resulting in apical parts completely covered by lanolin paste. The gynoecia were observed after 2 weeks and photographed under a dissecting scope.

## Results

To identify genes involved in gynoecium development that are expressed under the control of NGA factors, we compared the expression profiles in stage 8–13 dissected pistils from wildtype, quadruple *nga* mutants and plants overexpressing *NGA3* (stages defined after Smyth et al., 1990; Figures 1A,B). We selected transcripts with a fold change of >1.4 or <-1.4 and a corrected FDR *p*-value < 0.05. With those thresholds, we identified 1889 genes differentially expressed between wildtype and the quadruple *nga* mutant, 554 between wildtype and *35S::NGA3* and 637 between the quadruple *nga* mutant and *35S::NGA3*. Combining the results of the three comparisons, a list of 2449 genes were identified as putative targets of NGA regulation. With this final list we conducted a gene ontology analysis with the agriGO toolkit, finding that auxin-related genes were overrepresented in the dataset of differentially expressed transcripts (Suppl. Figure 1). Among them, genes related to auxin synthesis, transport, and response were identified, confirming previous reports of a functional relationship of NGA and auxin signaling (Alvarez et al., 2009; Trigueros et al., 2009), and suggesting that this interaction could occur at multiple levels.

To characterize in more detail how altered NGA activity influenced the spatial and temporal patterns of expression of their putative auxin-related targets, we introduced in *nga* mutants and in the *35S::NGA3* line reporter lines for several of these differentially expressed and other related genes, as well as markers for auxin accumulation.

### NGA mutations affect the expression of genes involved in auxin synthesis

**Figure 2:**
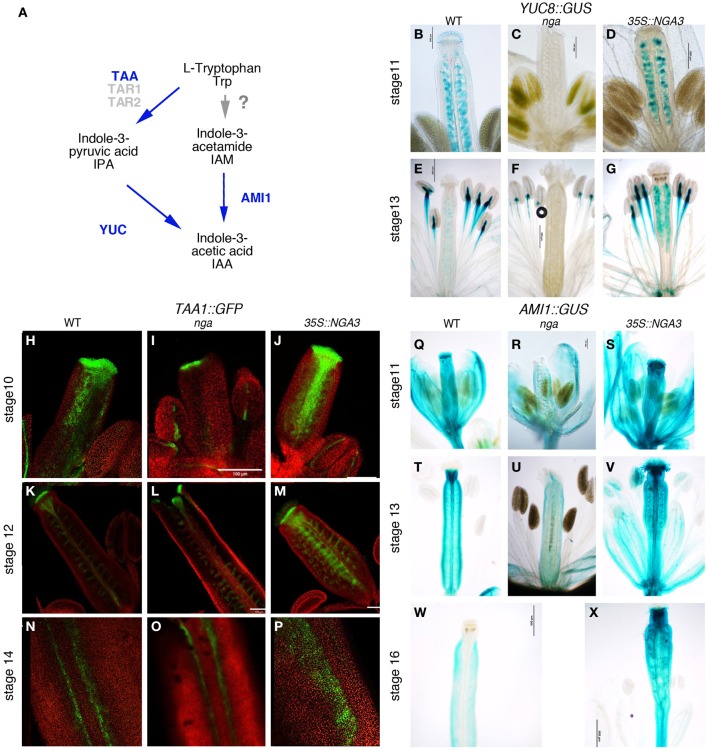
**Effect of NGA altered activity on the expression of genes involved in local auxin synthesis. (A)** Simplified scheme of the presumptive pathways for IAA biosynthesis studied in this work, adapted from Mano and Nemoto (2012). Genetic functions analyzed in this work are noted in blue. Question mark on the TRP>IAA pathway denotes that the conversion of TRP to IAM has not been demonstrated in plants **(B–G)** Histochemical detection of GUS activity driven by the *YUC8* promoter in wildtype **(B,E)**, *nga* quadruple mutants **(C,F)** and *35S::NGA3* gynoecia **(D,G)** at stage 11 **(B–D)** and at anthesis **(E–G)**. **(H–P)** *TAA1::GFP:TAA1* expression in wildtype **(H,K,N)**, *nga* quadruple mutants **(I,L,O)**, and *35S::NGA3* gynoecia **(J,M,P)** at stage 10 **(H–J)** and stage 12 **(K–M)**. **(N–P)** show close up views of the valve margins in the ovary region of anthesis pistils. **(Q–X)** Histochemical detection of GUS activity driven by the *AMI1* promoter in wildtype **(Q,T,W)**, *nga* quadruple mutants **(R,U)** and *35S::NGA3* gynoecia **(S,V,X)** at stage 11 **(Q–R)**, at anthesis **(T–V)**, and post-fertilization, at around stage 16 **(W–X)**.

It has been reported that *nga* mutations severely modify the expression patterns of *YUC2* and *YUC4* genes in the apical gynoecium (Trigueros et al., 2009). *YUC* enzymes catalyze the rate- limiting step in Trp-dependent auxin biosynthesis (Figure 2A) (Zhao et al., 2001; Mano and Nemoto, 2012). Only *YUC2* and *YUC4* have been shown to be strongly expressed in the apical developing gynoecium, suggesting that they could be essential contributors to auxin synthesis in this domain (Cheng et al., 2006). However, *yuc2 yuc4* double mutants show no evident phenotypes in floral development, indicating that other *YUC* genes may also be important to direct auxin synthesis in the pistil (Cheng et al., 2006). In the RNAseq dataset, the expression of one additional *YUC* gene, *YUC8*, was found to be strongly reduced in the quadruple *nga* mutant. In *YUC8::GUS* lines, expression could be observed in the ovules and in two small foci in the basal part of the style in stage 11–12 wildtype pistils (Figure 2B). This expression was completely absent in *nga* quadruple mutants (Figure 2C), while appeared unchanged or slightly increased in *35S::NGA3* pistils (Figure 2D). At later stages (13–15), expression in ovules was maintained in wildtype and *35S::NGA3* fruits (Figures 2E–G), although GUS activity was clearly stronger in the overexpression lines. These results confirm that *YUC8* is also upregulated by NGA factors at least in the apical gynoecium and likely in the ovules.

In addition to *YUC8*, RNAseq data revealed that expression of *TAA1* was also affected by *NGA* loss of function. *TAA1* encodes an enzyme that catalyzes the conversion of Trp to IPA, the proposed substrate for YUC enzymes (Mano et al., 2010; Stepanova et al., 2011). In wildtype developing gynoecia, a *TAA1::GFP:TAA1* reporter showed strong expression in the apical domain and in two longitudinal bands at medial positions in the developing gynoecial cylinder at stage 9–10 (Figure 2H). Later in development (st. 11), expression became restricted to a cell layer at the style/stigma junction and to the medial vascular bundles and the vascular veins of the funiculi (Figure 2K). In post-anthesis young fruits, expression was detected in the developing dehiscence zone at both sides of the replum (Figure 2N). *TAA1::GFP:TAA1* expression was reduced but not absent from the apical and medial domains of *nga* quadruple mutants at stage 8–9 (Figure 2I). In stage 11 *nga* gynoecia, since style and stigma do not form properly, the single cell layer of GFP expression below stigmatic cells could not be detected, but GFP accumulated at the tips of the valve protrusions and was mostly unchanged in other domains (Figures 2L,O). In *35S::NGA3* pistils, *TAA1::GFP:TAA1* expression was found in the same spatial pattern as in wildtype, although GFP signal appeared to be stronger in all domains and maintained for longer (Figures 2J,M). Thus, stage 11 *35S::NGA3* gynoecia still showed GFP signal in the medial region (Figure 2M), and in post-anthesis young *35S::NGA3* fruits, strong expression could be detected in the funiculi and expanding to the valves (Figure 2P).

*AMIDASE1 (AMI1)* encodes an enzyme that catalyzes the conversion of IAM to IAA and it has been proposed to contribute to auxin synthesis through an alternative pathway to the TAA1/YUC route (Figure 2A) (Mano et al., 2010). An *AMI1::GUS* reporter line showed *AMI1* promoter to be active in most floral organs. In the gynoecium, GUS activity could be detected at medium levels throughout the gynoecial tube, while in the style region it accumulated strongly from stages 10–11 (Figure 2Q). In anthesis flowers, GUS signal was very high in the style and it could also be detected in the vascular bundles (Figure 2T). Apical expression disappeared in developing fruits, while in the ovary, low levels of expression could still be detected (Figure 2W). In *nga* quadruple mutants, the strong expression in apical gynoecium typical of wildtype pistils was absent in preanthesis or anthesis pistils (Figures 2R,U). Conversely, *35S::NGA3* lines showed stronger GUS signal that was maintained in developing *35S::NGA3* fruits (Figures 2S,V,X).

### Auxin accumulation is reduced in the apical domain of the NGA mutant gynoecia

**Figure 3:**
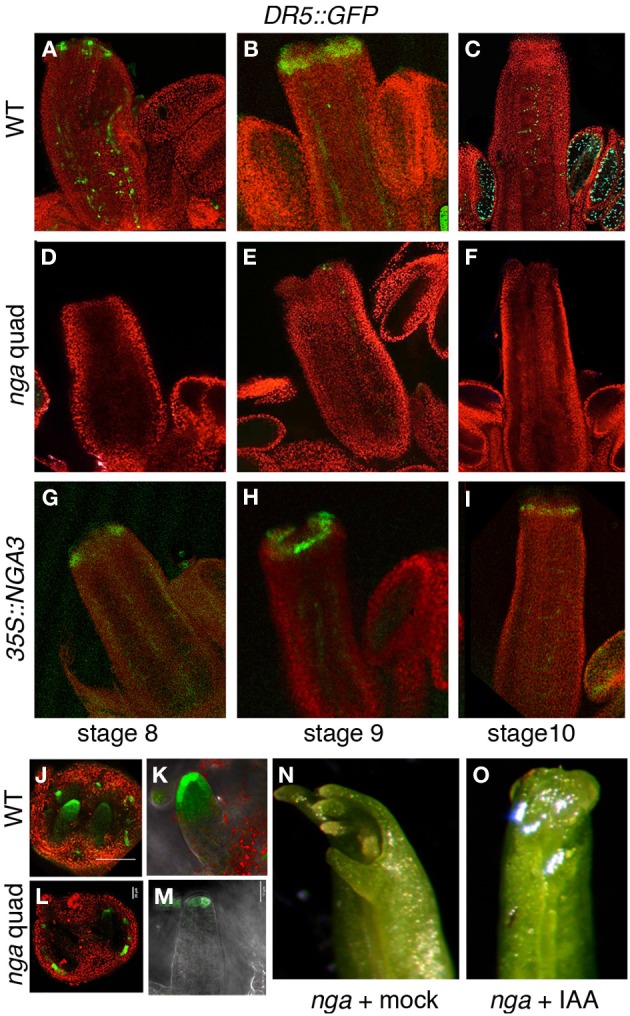
**Effect of NGA altered activity on *DR5rev::GFP* expression in gynoecium development. (A–C)** *DR5rev::GFP* in wildtype pistil development. **(A)** *DR5rev:GFP* is detected as discrete foci in stage 8 gynoecium **(B)** GFP is detected as a continuous apical ring in stage 9 gynoecium and in longitudinal strands at sites of main vascular development. **(C)** At stage 10, apical GFP expression is barely detected and signal is only clear in the funiculi of ovules. **(D–F)** *DR5rev::GFP* expression in *nga* quadruple mutants. GFP is virtually undetectable at stage 8 **(D)**, stage 9 **(E)**, or stage 10 **(F)** gynoecia. **(G–I)** *DR5rev::GFP* expression in *35S::NGA3* gynoecia. GFP expression is detected in similar patterns as wildtype in stage 8 **(G)** and stage 9 **(H)** gynoecia, while it appears to be slightly more persistent in the apical cells of stage 10 pistils **(I)**. **(J,K)** *DR5rev::GFP* expression in ovules of stage 10 wildtype **(J,K)** and nga quadruple mutants **(L,M)**. Note the reduced but detectable GFP activity at the tip of *nga* developing ovules. **(N,O)** Effect of micro-application of auxin to the apical end of *nga* mutant pistils. **(N)** Mock treated pistils, showing completely unfused apical ends with no signs of style or stigma development. **(O)** IAA-treated pistils show apical closure and very limited style development, but no stigmatic cells are able to develop.

Altogether, our results pointed to a greatly reduced or absent auxin synthesis in the apical domain of *nga* mutants and possibly a sustained increased auxin synthesis in the overexpression lines. If this was true, we could expect a reduced auxin accumulation in the apical domain of *nga* developing gynoecia and higher auxin levels in pistils and fruits. To test this hypothesis, we compared the activity of a *DR5rev::GFP* reporter in wildtype (Figures 3A–C), *nga* quadruple (Figures 3D–F) and *35S::NGA3* backgrounds (Figures 3G–I). *DR5rev::GFP* activity during Arabidopsis gynoecium development has been described (Benková et al., 2003; Girin et al., 2011; Marsch-Martinez et al., 2012a; Larsson et al., 2013). GFP expression is first detected as two lateral apical foci (stage 7), which at stage 8 also comprise two additional medial apical foci (Figure 3A), and at stage 9 extends as a continuous apical ring (Figure 3B). In *nga* gynoecia, the apical foci in stage 7–8 could be barely detected (Figure 3D), and the formation of the apical ring was never observed (Figure 3E). Surprisingly, in *35S::NGA* pistils, *DR5rev::GFP* activity was very similar to wildtype, indicating that in spite of the apparently increased auxin synthesis that could be expected from the stronger expression of *TAA1*, *YUC8*, or *AMI1*, the response of the reporter was not enhanced (Figures 3G–I). Interestingly, when *DR5rev::GFP* activity in ovule primordia of wildtype and *nga* mutants was compared, a reduction of GFP levels in *nga* ovules was observed, but not the absence of the distal auxin maxima (Figures 3J–M). This result suggested that, in spite of the absence of *YUC8* expression in *nga* ovules, the persisting expression of *YUC4* previously reported in this domain (Trigueros et al., 2009) was sufficient to direct auxin synthesis and allow ovule development.

Low auxin levels have been related to the *nga* phenotypes in style and stigma. Thus, reduced apical tissues were observed in a transgenic line where the *NGA3* promoter drove the expression of *iaaL,* a bacterial gene that encodes an enzyme that inactivates free auxin (Jensen et al., 1998; Trigueros et al., 2009). To test whether exogenous auxin treatments could restore style and stigma development, we performed micro-applications of IAA dissolved in droplets of lanolin to the tip of young developing *nga* gynoecia (stages 9–10). A limited partial rescue of the *nga* phenotypes was observed, with restored apical closure of the gynoecium, but no development of style or stigma typical cells, suggesting that the lack of auxin accumulation in this domain was probably not the only factor causing the *nga* phenotypes (Figures 3N,O).

In summary, auxin synthesis was likely very reduced in the apical domain of *nga* mutants, both through the TAA/YUC pathway and the presumptive AMI1 pathway, thus leading to low auxin levels in the distal region of *nga* developing pistils as deduced from the greatly reduced activity of the of *DR5rev::GFP* reporter. However, *NGA3* overexpression did not have a high impact in distal auxin accumulation, suggesting that NGA factors could also be interfering with other components of the auxin transport/response pathways.

### NGA mutations affect the expression of genes involved in auxin transport

**Figure 4:**
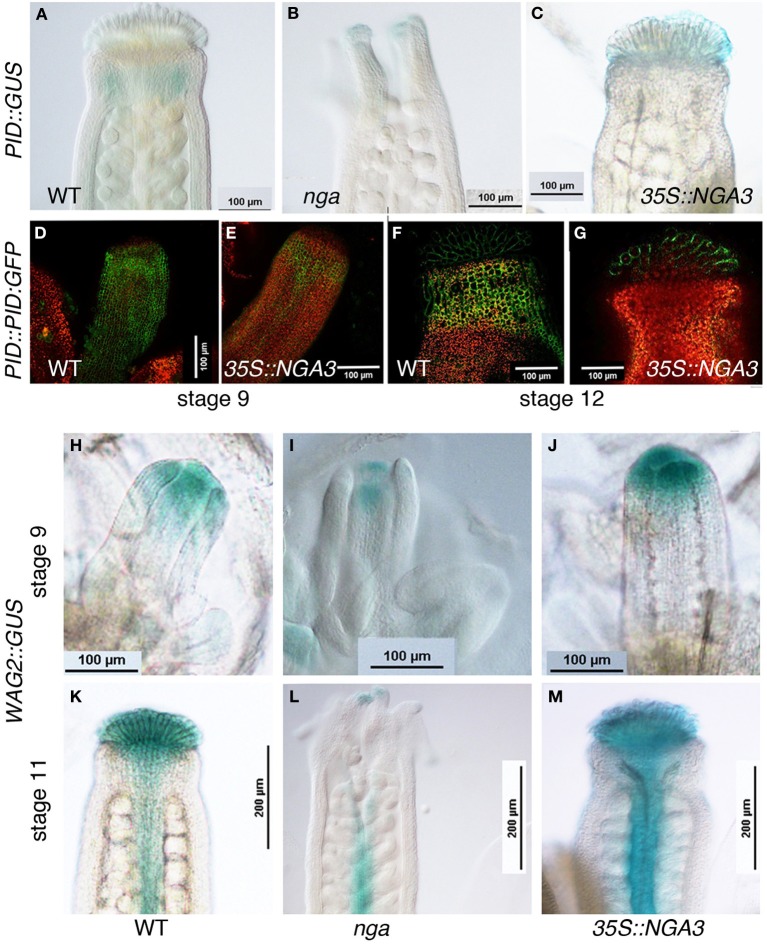
**Effect of NGA altered activity on the expression of protein kinases involved in the regulation of PIN subcellular polarization. (A–C)** Histochemical detection of GUS activity driven by the *PID* promoter in wildtype **(A)**, *nga* quadruple mutants **(B)** and *35S::NGA3* apical region of stage 12 gynoecia **(C)**. **(D–G)** *PID::PID:GFP* expression in stage 9 wildtype **(D)** or *35S::NGA3* gynoecia and in the style/stigma region of stage 12 wildtype **(F)** or *35S::NGA3* **(G)** pistils. **(H–M)** Histochemical detection of GUS activity driven by the *WAG2* promoter in wildtype **(H,K)**, *nga* quadruple mutants **(I,L)** and *35S::NGA3* **(J,M)** gynoecia at stage 9 **(H–J)** and stage 11 **(K–M)**.

Members of the PIN protein family of auxin efflux regulators have been shown to mediate various developmental processes, including carpel patterning. Polar, subcellular localization of PIN proteins determines the direction of auxin flux (reviewed in Friml, 2003) and this localization is partially regulated by their phosphorylation status, which depends on the antagonistic action of the PP2AA phosphatases, and kinases such as PINOID, WAG1, WAG2, and PID2 (Santner and Watson, 2006; Michniewicz et al., 2007). *PID* and *WAG2* expression has been reported in apical tissues of Arabidopsis developing gynoecia (Girin et al., 2011). Moreover, *pid* mutants show severe carpel patterning phenotypes similar to those found in *pin1* or *pin3 pin7* mutants (Okada et al., 1991; Bennett et al., 1995; Benková et al., 2003). RNAseq analyses revealed altered expression levels of both *PID* and *WAG2* in either *nga* or *35S::NGA3* pistils, and therefore, we introduced *PID* and *WAG2* reporters into these backgrounds. *PID::GUS* activity was weakly detected in the style of stage 11 wildtype carpels (Figure 4A). In *nga* mutants, expression could still be detected in the apical protrusions typical of *nga* gynoecia (Figure 4B), while in *35S::NGA3* pistils, expression was absent in the style but present in the stigma (Figure 4C). Moreover, a *PID::PID:GFP* reporter line showed a substantially reduced GFP signal in *35S::NGA3* lines when compared to wildtype, suggesting that NGA3 could be preventing PID accumulation (Figures 4D–G). *WAG2::GUS* showed early expression in the distal end of the stage 9 gynoecial tube in wildtype, *nga*, or *35S::NGA3* (Figures 4H–J). This apical expression was maintained until stage 11 in wildtype or *35S::NGA3* pistils (Figures 4K,M), while clearly reduced in *nga* mutants (Figure 4L). These results suggested that NGA factors could regulate *PID* and *WAG2* in opposite directions, repressing *PID* while activating *WAG2*, similarly to what it has been described for the bHLH transcription factors INDEHISCENT and SPATULA (Girin et al., 2011).

**Figure 5:**
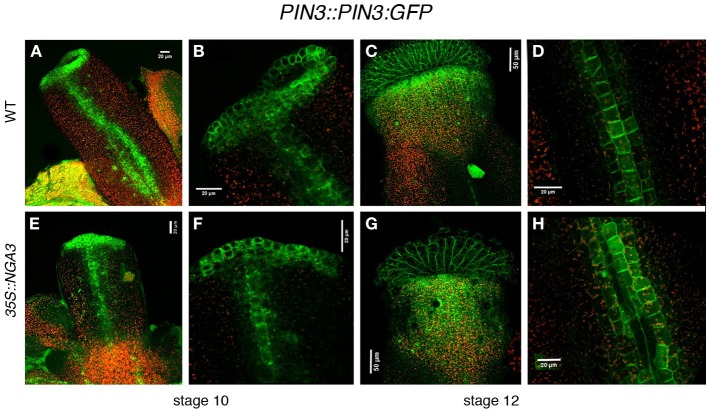
**Effect of NGA altered activity on PIN3 protein localization**. *PIN3::PIN3:GFP* expression was observed in wildtype **(A–D)** and *35S::NGA3* **(E–H)** developing gynoecia at stage 10 **(A,B,E,F)** and stage 12 **(C,D,G,H)**. Close view of the apical ring in wildtype **(B)** or *35S::NGA3* **(F)** stage 10 pistils did not show a clear PIN3 subcellular polarization, although in the longitudinal stripes of cells running along the ovary, PIN3 appears to be predominantly at the basal side of cells. **(C)** Stage 12 wildtype gynoecia showed strong GFP signal in stigmatic cells and in a domain in the style just below the stigma. **(D)** In wildtype stage 12 ovaries three rows of cells showed PIN3:GFP expression, where PIN3:GPF protein appeared to be localized at the basal side of cells. **(G)** In *35S::NGA3* stage 12 pistils, PIN3:GFP is detected in stigma and a broader domain of the style **(H)** In *35S::NGA3* stage 12 ovaries, PIN3:GFP expands to 4–5 cell longitudinal rows, also apparently localized to the basal side of cells.

The differences in *PID* and *WAG* expression caused by altered NGA activity suggested that PIN protein localization could also be affected. PIN1 protein localization has been described in developing fruits, but no detailed patterns of expression have been described for any of the PIN transporters throughout gynoecium development (Sorefan et al., 2009). We compared *PIN1::PIN1:GFP* in wildtype, *nga*, and *35S::NGA3* backgrounds, but no clear differences could be observed (Suppl. Figure 2). Likewise, the *PIN7::PIN7:GFP* reporter line available to us showed very low levels of GFP activity and we could not obtain conclusive results. Finally, we determined *PIN3::PIN3:GFP* expression in wildtype developing gynoecia. PIN3:GFP protein was localized in a narrow apical ring and two longitudinal stripes at epidermal medial positions in stage nine pistils (Figures 5A,B). Apical expression was maintained at stage 12, restricted to the stigma and the underlying layers in the style, and also in the replum domain, although at lower levels (Figures 5C,D). It was difficult to determine the subcellular orientation of PIN3 both in the apical ring and in the replum domain at early stages, and therefore the direction of the auxin flux was not easily deduced. At stage 12, however, PIN3:GFP protein was mostly localized in the basal side of cells in the style and the replum, suggesting that auxin flux would be directed toward the basal part of the ovary (Figure 5D). We failed to introduce the *PIN3::PIN3:GFP* reporter in the *nga* quadruple mutants background, but PIN3:GFP accumulation was studied in the *35S::NGA3* background. *35S::NGA3* pistils showed similar accumulation patterns of PIN3 at stage 9 (Figures 5E,F). However, from stage 11, PIN3 accumulation appeared to be increased both in the apical domain and the replum region, where it comprised a higher number of cell rows, suggesting that basipetal auxin flux could be facilitated in the *35S::NGA3* background (Figures 5G,H).

### NGA mutations affect in different ways the expression of auxin response factors (ARFs) throughout gynoecium development

Finally, we took advantage of the recently created collection of ARF reporters described by Rademacher et al. (2011) to examine the effect of NGA altered activity on the protein expression patterns of several ARFs expressed in the apical domain of developing gynoecia, namely ARF1, ARF8, ARF11, and ARF18. We also included in our analyses ARF3/ETT, since it was also expressed in the developing pistil, but we did not observe significant changes in reporter activity in the *nga* or *35S::NGA3* backgrounds (Suppl Figure 3).

**Figure 6:**
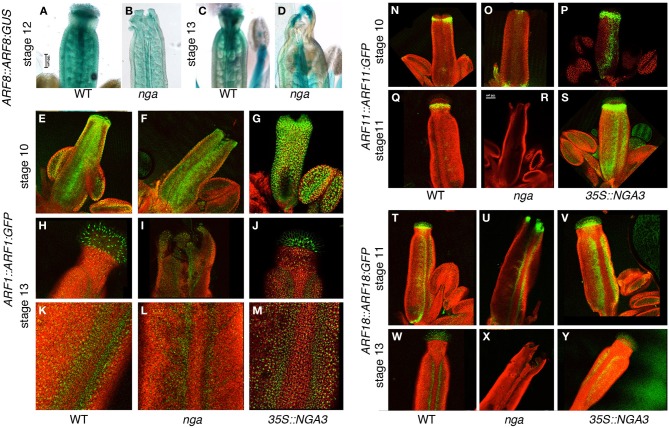
**Effect of NGA altered activity on the expression of ARFs throughout gynoecium development. (A–D)** Histochemical detection of *ARF8::ARF8:GUS* activity in wildtype **(A,C)**, and *nga* quadruple mutants **(B,D)** gynoecia at stage 12 **(A,B)** and at anthesis **(C,D)**. While in stage 12 pistils ARF8 apical accumulation is clearly reduced **(B)**, at anthesis shows some expression at valve protrusions formed in the nga quadruple mutant **(D)**. **(E–M)** *ARF1::ARF1:GFP* expression in wildtype **(E,H,K)**, *nga* quadruple mutants **(F,I,L)** and *35S::NGA3* gynoecia **(G,J,M)** at stage 10 **(E–G)** and at anthesis **(H–M)**. **(H–J)** show close up views of the apical domain, and **(K–M)** show close up views of valve margins in the ovary region of anthesis pistils. **(N–S)** *ARF11::ARF11:GFP* expression in wildtype **(N,Q)**, *nga* quadruple mutants **(O,R)** and *35S::NGA3* gynoecia **(P,S)** at stage 10 **(N–P)** and at stage 11 **(Q–S)**. Note the strongly reduced GFP signal in the apical region of *nga* mutants **(O,R)** and the strong signal associated with valve margins of *35S::NGA3* developing gynoecia **(P,S)**. **(T–Y)** *ARF18::ARF18:GFP* expression in wildtype **(T,W)**, *nga* quadruple mutants **(U,X)** and *35S::NGA3* gynoecia **(V,Y)** at stage 11 **(T–V)** and at anthesis **(W–Y)**. Note expanded GFP signal associated with valve margins of *35S::NGA3* pistils **(V,Y)**.

*ARF8::ARF8:GUS* reporter activity in wildtype gynoecium development has been already described (Goetz et al., 2006). ARF8 protein appears strongly associated to transmitting tissues, specially stigma and transmitting tract, and, at lower levels, in the ovary walls and the ovules (Figures 6A,C). Loss of *NGA* function mainly affected the accumulation of ARF8:GUS in the apical end of the gynoecium, which appeared reduced at anthesis although maintaining foci of expression at the apical end of valve protrusions, while did not alter significantly ARF8:GUS levels in the ovary or the ovules (Figures 6B,D). Crosses between *ARF8::ARF8:GUS* and *35S::NGA3* line failed and therefore we were not able to analyze the activity of the reporter in this background for this work.

*ARF1::ARF1:GFP* reporter showed activity in medial and epidermal tissues of stage 10 wildtype gynoecia, with higher levels of GFP signal at the apical end of the gynoecial tube (Figure 6E). In anthesis wildtype pistils, signal was mainly associated with stigmatic cells and valve margins, with low but consistent accumulation of ARF1:GFP detected in the epidermal cells of style and valves (Figures 6H,K). In *nga* mutants, ARF1:GFP patterns were very similar to wildtype in preanthesis and anthesis stages, although likely due to the lack of stigmatic cells, no strong signal was detected in apical cells of anthesis *nga* gynoecia (Figures 6F,I,L). As for *35S::NGA3* lines, ARF1:GFP expression was found at similar domains, although it appeared to be increased in level (Figures 6G,J). This stronger expression was more conspicuous at anthesis, where valves showed clearly enhanced fluorescent signal (Figure 6M).

*ARF11::ARF11:GFP* reporter activity was detected already at stage 9–10 in the presumptive developing style and the valve margins (Figure 6N), accumulating below the stigmatic cells until stage 12 (Figure 6Q), and becoming barely detected at anthesis and later stages. In *nga* quadruple mutants, ARF11:GFP protein could be only detected at very reduced levels in a small apical domain of stage 10 pistils (Figures 6O,R), while in *35S::NGA3*, ARF11:GFP protein accumulated similarly to wildtype in the apical tissues although in an expanded domain (Figure 6P). Interestingly, and unlike from wildtype, ARF11:GFP accumulated in the valve margins of *35S::NGA3* gynoecia from early stages of development, where it could still be strongly detected prior and at anthesis (Figures 6P,S).

*ARF18::ARF18:GFP* reporter drove a strong GFP signal in the apical domain of stage 10 wildtype pistils (style and stigmatic cells) and at the valve margins (Figure 6T). At later stages (stage 13 and postanthesis), ARF18:GFP could still be detected in the stigmatic cells and the differentiating dehiscence zones, restricted to a few cell rows (Figure 6W). In *nga* quadruple mutants, ARF18:GFP accumulation was similar to that observed in wildtype, in spite of the absence of style and stigma, and signal was detected in the valve protrusions that developed apically. In stage 11 *nga* gynoecia, a strong GFP signal could be observed in the apical domain and weakly at the valve margins (Figure 6U). Apical expression could still be weakly detected in anthesis *nga* pistils, to become restricted to the dehiscence zones in post-anthesis stages (Figure 6X). *35S::NGA3* pistils also showed similar patterns of ARF18:GFP accumulation in apical domains and in the valve margins (Figure 6V). In anthesis and post-anthesis stages, however, the accumulation of ARF18:GFP was found in a broader area at the valve margins, correlating with the lateral expansion of the dehiscence zones in *35S::NGA3* fruits (Figure 6Y) (Trigueros et al., 2009).

## Discussion

The study carried out in this work shows how alterations in NGA function have significant effects in auxin signaling throughout gynoecium development and that these interactions likely occur at multiple levels.

First, we have shown that the apical auxin maxima that forms in stage 8–9 wildtype gynoecia cannot be detected in *nga* quadruple mutants. While it is not conclusively proven that this maxima is directly responsible for style and stigma differentiation, it is clearly temporally correlated with the development of these tissues. Moreover, the inability of *nga* mutants to form this maxima and to differentiate apical tissues that we show in this work, together with the *nga*-like phenotypes of lines where the *NGA3* promoter directed the expression of *iaaL*, an enzyme that inactivates the pool of active auxin (Jensen et al., 1998), supports this direct causative link. On the other hand, the partial rescue of *nga* apical defects by local auxin application might suggest that the absence of auxin maxima is not the only cause of *nga* phenotypic defects. However, the method that we used for local auxin treatment is coarse and may not reproduce properly the spatial distribution or the timing of auxin accumulation dynamics, therefore providing a partial picture of the expected effects and limiting the validity of these conclusions. Interestingly, the generation of auxin maxima as revealed by *DR5rev::GFP* in developing gynoecia that constitutively express *NGA3* is not significantly different from that of wildtype. *35S::NGA3* pistils do not show a dramatic overproliferation of style and stigma, thus indirectly reinforcing the idea of the putative instructive role of auxin accumulation in these tissues to direct the development of apical tissues (Trigueros et al., 2009).

The likely failure to accumulate auxin in the apical domain of *nga* mutants can also explain the insensitivity of *nga* mutants to PAT inhibition (Alvarez et al., 2009): since no auxin is present, it can be expected that no basipetal transport takes place and therefore, no phenotypic defects result from this inhibition. It has been shown that *shi/sty* mutants are hypersensitive to NPA treatment (Ståldal et al., 2008). This situation is opposite to that found in *nga* mutants, in spite of the almost identical phenotypes found in gynoecium development and the apparent convergent regulation of YUC-mediated auxin synthesis by both NGA and SHI/STY factors (Kuusk et al., 2006; Sohlberg et al., 2006; Trigueros et al., 2009; Eklund et al., 2010). This could reflect a different role of NGA and SHI/STY factors in the establishment of auxin maxima or in the regulation of downstream effectors in response to those. To understand these mechanistical differences, it would be useful to describe auxin accumulation throughout gynoecium development in *shi/sty* multiple mutants.

Local auxin synthesis appears to be strongly reduced in apical tissues of *nga* mutants. It already has been shown that *NGA* downregulation leads to the loss of *YUC2* and *YUC4* activation in the apical domain of developing gynoecia (Trigueros et al., 2009). We show here that *YUC8* expression is completely absent in *nga* mutant pistils, while slightly increased in *35S::NGA3* lines. It could be envisioned that the lack of apical auxin maxima in *nga* pistils could be due to the absence of YUC-mediated auxin synthesis in this domain. The phenotypes of *yuc2 yuc4 yuc8* triple mutants have not been described, so it is not possible to directly compare both scenarios. Even in the triple mutants, since *YUC2* and *YUC4* are normally expressed in *nga* mutants outside the apical gynoecium, loss of *YUC2* and *YUC4* function could have additional effects that might obscure the specific role of *YUC2/4/8* in style and stigma differentiation, so in addition to generating and characterizing the *yuc2 yuc4 yuc8* triple mutants, it might be necessary to inactivate specifically all three enzymes in the apical developing gynoecium. In addition to the effect of *nga* mutations on *YUC* gene expression, *TAA1* and *AMI1* also appear to be under NGA direct or indirect regulation. TAA1 has been recently placed in the same biosynthetic route as the YUC enzymes (Stepanova et al., 2011) and thus it would be possible that the moderate effects of NGA altered function in *TAA1* expression would not lead to dramatic differences in auxin synthesis rates through this TAA-YUC pathway. Unlike the *YUC* genes for which detailed expression patterns in carpels have been reported, *TAA1* is expressed in carpel margins of stage 12 and postanthesis wildtype pistils. Interestingly, this valve margin expression is reduced in *nga* mutants while increased in *NGA3* overexpressors. While the role of this putative local auxin synthesis at the valve margin is currently unknown, as well as the precise role of *NGA* in valve margin development, the possible altered auxin synthesis in valve margins in response to NGA differential activity could partly explain the changes in the expression levels of *ARF1*, *ARF11* or *ARF18* in the different *NGA* backgrounds revealed in this study. Finally, a third putative contributor to local auxin synthesis is the AMI1 enzyme, which catalyzes the transformation of IAM to IAA. It is still unclear whether AMI1 activity significantly contributes to auxin synthesis in inflorescence development (Mano et al., 2010; Zhao, 2010), but the strong *AMI1* expression in developing gynoecia and, specially, in apical and transmitting tissues, suggests that it may have a role in auxin production in this domain. We show here that *nga* mutations significantly reduce *AMI1* accumulation in the apical pistil, while *NGA3* overexpression leads to increased and persistent levels of *AMI1* expression, thus indicating that NGA could also positively regulate AMI1 activity in these tissues and hence putative auxin synthesis through this pathway. It has been described that *ami1* mutants (aka *attoc64-I*) do not show phenotypic defects (Aronsson et al., 2007), which could be due to redundancy with other members of the family, and therefore is its premature to speculate at this point about the relevance of the NGA-AMI1 functional relationship. However, the convergent effect of *NGA* mutations on the regulation of *TAA1*, *YUC,* and *AMI1* strongly suggests that NGA factors may function as strong positive regulators of auxin synthesis in the apical gynoecium.

The strongly reduced or absent local auxin synthesis in the apical developing *nga* mutant gynoecia probably contributes to the reduced auxin accumulation observed in these tissues, although it is unlikely to be the only cause. It is generally accepted that auxin maxima are mainly produced by PAT (Grieneisen et al., 2013), and there are examples of these maxima directing auxin synthesis that could reinforce auxin accumulation patterns (Grieneisen et al., 2007). In this work, it has been shown how enzymes involved in auxin synthesis are still expressed in the apical gynoecium after the *DR5rev::GFP* reporter signal has faded or is very reduced, suggesting that additional mechanisms have also an impact in auxin distribution downstream auxin synthesis. Clearly more work would be needed to resolve the interplay between transport, synthesis and probably other components of the pathway. Such further work should include a detailed characterization of auxin flux as directed by auxin transporters such as several PIN-family members or other transporters. Unfortunately, our analyses on the effect of *NGA* loss or gain of function on PIN proteins have not produced clear conclusions. Still, our results indicate that auxin transport is likely altered in *nga* mutants or the *35S::NGA3* line, since the expression of *PID* and *WAG2*, major regulators of PIN polarization, as well as the expression domain of PIN3 are affected by *NGA* altered function. In this sense, the expanded domain of expression of PIN3 observed in *35S::NGA3* pistils could facilitate auxin depletion from the apical domain through increased basipetal auxin transport, thus providing a hint on the mechanisms that could explain the wildtype-like response of *DR5rev::GFP* observed in *35S::NGA3* developing gynoecia.

Finally, protein accumulation patterns for several ARFs expressed through gynoecium development have been described in wildtype, *nga* mutants and *35S::NGA3* lines. We have found that NGA factors appear to differentially regulate the accumulation of the different ARFs under study. Thus, the apical domain of accumulation found for ARF11 is completely lost in *nga* mutants, while apical expression of *ARF8* and *ARF1* (and only slightly that of *ARF18*) are reduced, but not absent, from *nga* mutant apical gynoecia. Interestingly, *NGA3* constitutive expression appears to induce the expanded expression at valve margins of *ARF1*, *ARF11*, and *ARF18*, similarly to what was observed for *TAA1* expression, suggesting that they might be responsive to local auxin synthesis putatively mediated by TAA1. It has been described that *35S::NGA3* fruits have enlarged dehiscence zones and thus, this expanded expression domains could be also due to an indirect effect of *NGA3* overexpression on fruit morphology. Because no function has been assigned yet to *ARF1*, *ARF11*, or *ARF18* in gynoecium development, it remains to be studied whether ARF regulation may mediate *NGA* functions in this process.

In summary, our work shows that NGA factors impact on auxin signaling pathways at multiple levels throughout pistil development. First, and more importantly, NGA factors appear to be essential, but not sufficient for auxin synthesis in the apical developing gynoecium, since several members of the *YUC* family, as well as *TAA1* and *AMI1* were not expressed in this domain in *nga* quadruple mutants, but only showed moderately increased expression in *35S::NGA3* lines. Accordingly, *DR5rev::GFP* showed no activity in *nga* mutants but no significant differences in *35S::NGA3* pistils when compared to wildtype. It is thus tempting to speculate that NGA could only direct auxin synthesis in the presence of other factors, for which SHI/STY family members are strong candidates. In addition, NGA altered activity affected the expression of *PID* and *WAG2*, regulators of PIN subcellular localization, and thus likely had an impact on auxin transport in parallel to the effect on auxin synthesis. Finally, protein accumulation in pistils of several ARFs was differentially affected by *nga* mutations or *NGA* overexpression, suggesting that these accumulation patterns depend not only on auxin distribution but could be also regulated by transcriptional networks involving NGA factors. Again, *NGA3* constitutive expression did not result in wide activation of ARF expression in the gynoecium, reinforcing the idea of NGA requiring additional factors to exert their regulatory functions.

### Conflict of interest statement

The authors declare that the research was conducted in the absence of any commercial or financial relationships that could be construed as a potential conflict of interest.
