# Supplementary material for: The effect of NGATHA altered activity on auxin signaling pathways within the Arabidopsis gynoecium
Source: Front Plant Sci. 2014 May 21;5:210. doi: 10.3389/fpls.2014.00210 (PMC4033193; doi:10.3389/fpls.2014.00210)
Supplement: Supplementary file 1 [file DataSheet1.DOCX]

**Supplementary Material**


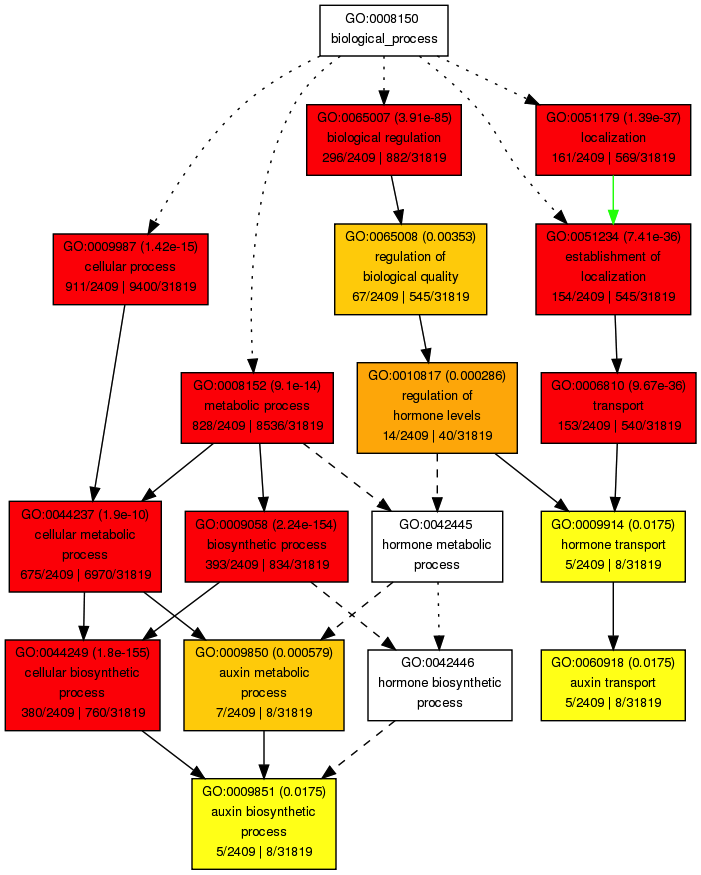

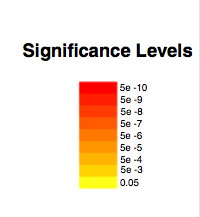


Supplementary figure 1. GO Slim Plant term enrichment of genes with altered expression expression in *nga/35S::NGA3* backgrounds related to auxin signaling pathways. GO term IDs are indicated, p-values in brackets**.** Molecular Function. Color Scale represents FDR adjusted p-values < 0.05. Solid, dashed, and dotted lines represent either two, one, or zero enriched terms at either end of the line.


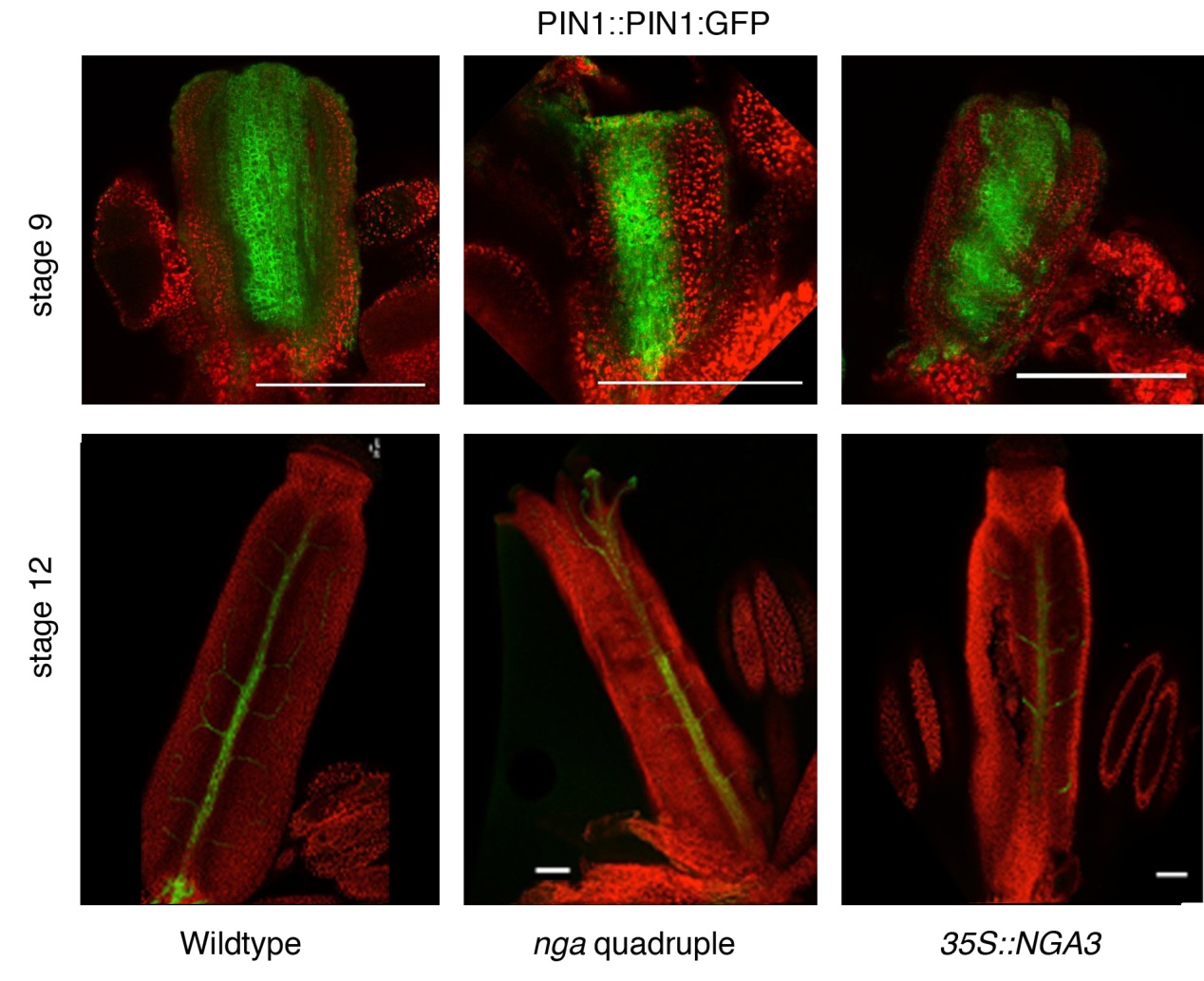


Supplementary figure 2. Effect of NGA altered activity on PIN1 protein localization. *PIN1::PIN1:GFP* expression was observed in wildtype (left), *nga* (center) and *35S::NGA3* (right) developing gynoecia at stage 9 (top panels) and stage 12 (bottom panels). No significant changes are observed in PIN1:GFP protein distribution.


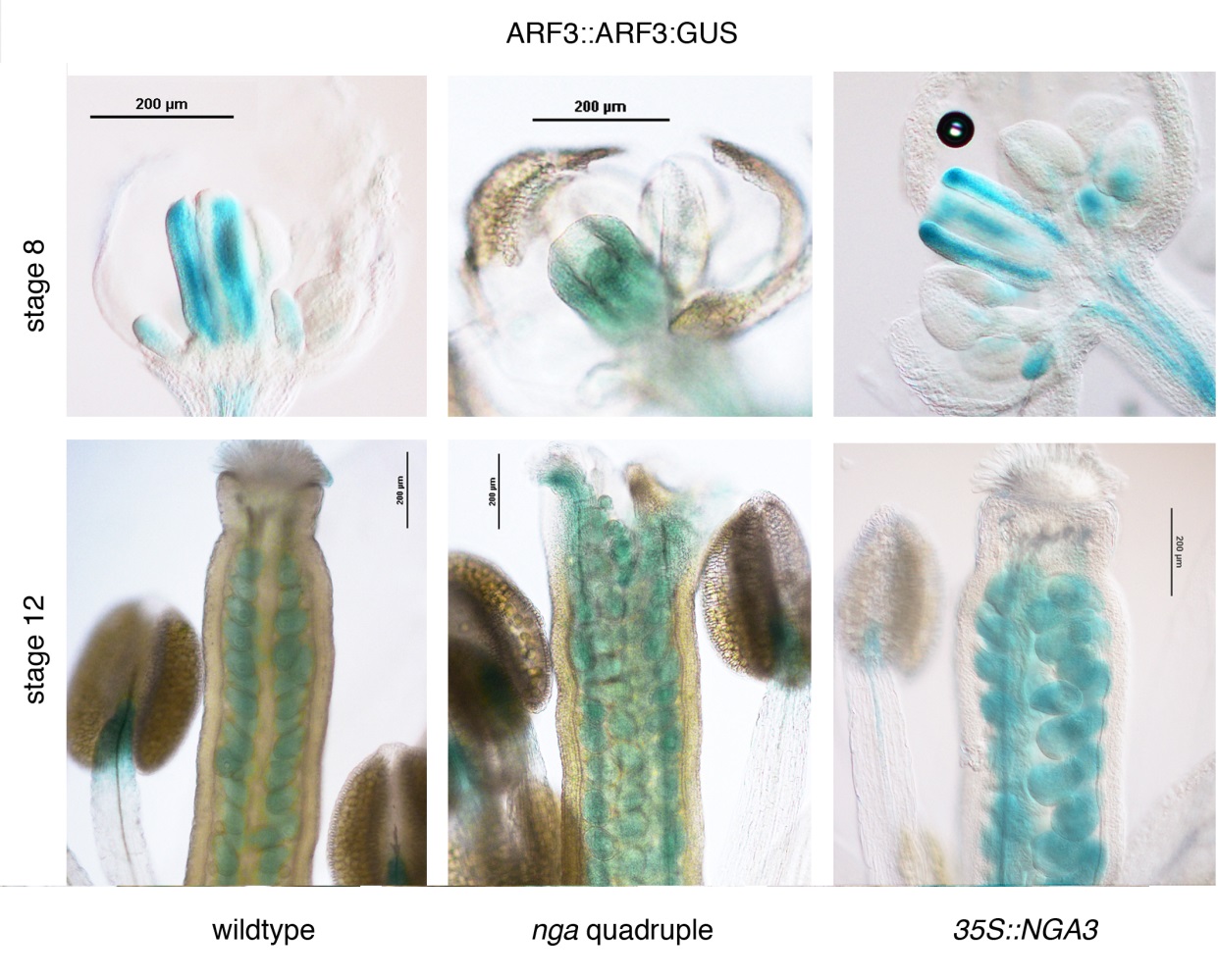


Supplementary figure 3. Effect of NGA altered activity on ARF3/ETT protein localization. *ARF3::ARF3:GUS* expression was observed in wildtype (left), *nga* (center) and *35S::NGA3* (right) developing gynoecia at stage 8 (top panels) and stage 12 (bottom panels). No significant changes are observed in ARF3:GUS activity.
